# Determination of silver nanoparticle release from antibacterial fabrics into artificial sweat

**DOI:** 10.1186/1743-8977-7-8

**Published:** 2010-04-01

**Authors:** Kornphimol Kulthong, Sujittra Srisung, Kanittha Boonpavanitchakul, Wiyong Kangwansupamonkon, Rawiwan Maniratanachote

**Affiliations:** 1 National Nanotechnology Center, National Science and Technology Development Agency, 111 Thailand Science Park, Phahonyothin Road, Pathumthani, 12120, Thailand; 2 Department of Chemistry, Faculty of Science, Srinakharinwirot University, Sukhumwit 23, Bangkok, 10110, Thailand

## Abstract

Silver nanoparticles have been used in numerous commercial products, including textiles, to prevent bacterial growth. Meanwhile, there is increasing concern that exposure to these nanoparticles may cause potential adverse effects on humans as well as the environment. This study determined the quantity of silver released from commercially claimed nanosilver and laboratory-prepared silver coated fabrics into various formulations of artificial sweat, each made according to AATCC, ISO and EN standards. For each fabric sample, the initial amount of silver and the antibacterial properties against the model Gram-positive (*S. aureus*) and Gram-negative (*E. coli*) bacteria on each fabric was investigated. The results showed that silver was not detected in some commercial fabrics. Furthermore, antibacterial properties of the fabrics varied, ranging from 0% to greater than 99%. After incubation of the fabrics in artificial sweat, silver was released from the different fabrics to varying extents, ranging from 0 mg/kg to about 322 mg/kg of fabric weight. The quantity of silver released from the different fabrics was likely to be dependent on the amount of silver coating, the fabric quality and the artificial sweat formulations including its pH. This study is the unprecedented report on the release of silver nanoparticles from antibacterial fabrics into artificial sweat. This information might be useful to evaluate the potential human risk associated with the use of textiles containing silver nanoparticles.

## Background

A growing interest in nanotechnology has lead to the increased production and application of nanoparticles worldwide. They are incorporated into various categories of consumer products, including cosmetics, textiles, electronics and medicines [1,2]. Silver nanoparticles are potent and broad-spectrum antibacterial agents with activity against diverse species within both Gram-positive and Gram-negative bacteria [3]. They show a good antibacterial property with their large surface area to volume ratio, which provides a better contact with the microorganism [4]. Silver nanoparticles have been applied in diverse consumer products, such as clothes, socks and laboratory gowns, as well as in medical products, such as surgical gowns and dressing bandages, which are claimed to have the ability to inhibit bacterial growth [5,6]. Concerning their safety, the Environmental Protection Agency (EPA) published a notice for public review and a filed petition, open to comment by concerned parties. That petition requested EPA to classify and regulate all products containing nanoscale silver as pesticides by taking regulatory actions under the Federal Insecticides, Fungicide and Rodenticide Act (FIFRA), as well as analyze the potential human health and environmental risks of nanoscale silver [7]. However, those issues will potentially affect many stakeholders and, therefore, need public response and comment.

Exposure to these materials during manufacturing and application can occur via ingestion, inhalation and dermal contact. In humans, it is well known that long-term ingestion of silver compounds can cause irreversible skin discoloration or Argyria [8]. The permissible exposure limit recommended by the National Institute for Occupational Safety and Health is 0.01 mg/m^3 ^for all forms of silver [9]. However, in rats, inhalation of silver nanoparticles caused adverse effects to the liver, kidney and lungs as well as the silver being observed to accumulate in several tissues [10,11], and thus nanosilver exposure may need a separate review and revision for human and environmental exposure levels.

The most common route of exposure to silver nanoparticles from antibacterial clothing textiles is via skin contact. Even though human skin consists of several semi-permeable to impermeable layers, and so provides a good protective barrier, it has been reported that silver nanoparticles can penetrate through the skin [12]. Adverse effects on human health might occur since several investigations have indicated that silver nanoparticles were toxic to many mammalian cultured cells [13,14]. In addition, among several metal nanoparticles, silver was found to be the most toxic to germ line stem cells [15]. The toxicity of silver nanoparticles, however, also depends upon their surface chemistry [16].

Recently, it was reported that silver nanoparticles are released into the aquatic environment during the washing process of silver-treated fabrics [17]. In relation to that investigation, other research groups reported that silver nanoparticles exerted toxic effects on aquatic organisms by reducing the algal photosynthetic yield and inducing abnormalities in Zebrafish embryos [18,19]. Therefore, the ultimate release load and release rate of silver nanoparticles from commercial products during their life cycle into the environment might present a potential risk to the ecological system.

Despite that clear risk, however, information of how many silver particles can be released from impregnated fabric products during wearing is still lacking. Artificial sweat is an *in vitro *chemical test model that is used for assaying metal release from products intended to be in close contact with the skin. They have been used in several studies of silver and nickel release from jewelry [20,21]. The aim of this study was to determine the degree of release of silver particles from nano-silver treated fabrics into artificial sweat, using four different preparations from AATCC, ISO and EN standards, so as to provide a pH range from 4.3 to 8.0. The silver content was measured using a graphite furnace atomic absorption spectroscopy (GFAAS). The antibacterial properties of the fabrics against Gram-positive and Gram-negative bacteria were also investigated.

## Methods

### Chemicals

Lactic acid was purchased from Carlo Erba (Rodano, Italy). L-histidine monohydrochloride monohydrate was obtained from Acros Organics (Geel, Belgium). Disodium hydrogen orthophosphate dodecahydrate, sodium dihydrogen orthophosphate dehydrate and sodium phosphate dibasic anhydrous were purchased from Fluka (Buchs, Germany). A commercial "Sanitized^® ^T27-22 Silver", herein referred to as "silver suspension", was obtained from Clariant (Burgdorf, Switzerland). According to the product's information, this reagent is composed of silver chloride and titanium dioxide suspended in an aqueous solution at an approximate total density of 1 g/mL. Non-ionic surfactant (Serwet^® ^Din), a secondary alcohol ethoxylate surfactant, was obtained from Winimex Industry (Samut Prakan, Thailand). Tryptic soy broth (TSB) and Tryptic soy agar (TSA) were purchased from BD (Franklin Lakes, NJ). All other chemicals were of the highest grade available.

### Sample preparation

In this study, the amount of silver released from five laboratory-prepared fabrics (samples A0, A1, A2, A3 and A4) and six commercial fabrics (samples B, C, D, E, F and G) was investigated. Laboratory fabrics were pretreated by immersing (21 × 29.7 cm^2^) of cotton fabric in 1 L of 0.2% (w/v) non-ionic surfactant and 0.2% (w/v) sodium hydroxide and then heated at 95°C for 30 min. The cotton fabrics were then cooled with water and allowed to dry at room temperature. For silver coating, these fabrics were treated with the silver suspension at five concentrations of 0, 0.5, 1, 5 and 10 g/L, for samples A0, A1, A2, A3 and A4, respectively, by using a Laboratory Padder (model PA-U, Newave Lab Equipments, Taiwan) with 80% wet pick-up of the fabric weight, and then dried at 100°C for 1 min in a Laboratory Stenter (model M-3, Mingscape International, Taiwan). Finally, the fabrics were cured at 120°C for 3 min in the same machine. Samples B, C, D, E, F and G were claimed as nanosilver shirts by, and purchased from, six different manufacturers in Thailand. Three parts, one each from the front, back and arm, of commercial shirts were selected for further experiments.

### Characterization of silver nanoparticles

A small piece of fabric was placed into a tightly closed crucible and burned in a furnace (CWF1000, Carbolight, UK) at 600°C for 3 h and then allowed to cool down at room temperature. The fabric ash was collected and dispersed in deionized water. Silver nanoparticles in the silver suspension (Sanitized^® ^T27-22 Silver) and the fabric ash were then visualized by a scanning electron microscopy (SEM; SRS-3400N, Hitachi, Japan). An energy-dispersive X-ray analysis (EDX) was used to confirm the presence of silver particles.

### Determination of the lower detection limit of GFAAS

The lower detection limit of GFAAS was determined according to the URACHEM Guide-1998, with some modification. In brief, 10 samples of 5% (v/v) nitric acid solution were independently prepared and subjected to the GFAAS. The detection limit of this machine, calculated from mean sample value plus three times of standard deviation, was found to be 0.26 μg/L.

### Measurement of initial silver content

Fabric samples, weighing between 0.20 - 0.30 g, were digested by a microwave digestion system (MARE, CEM Corp., Matthews, NC) in 5 ml of 14.4 M nitric acid to break down the fabric fibers and dissolve all the silver content. The microwave irradiation cycles were 250 W for 5 min, 400 W for 5 min and 600 W for 5 min. The digested fabrics were then cooled, and diluted up to 25 ml with deionized water to enable quantification of silver by a graphite furnace atomic absorption spectroscopy or GFAAS (Perkin Elmer Analyst 300, Waltham, MC). The fabrics which had no detectable silver were further subjected to an Inductively Coupled Plasma Spectrometer (ICP, Varian 730-ES, South San Francisco, CA) to confirm the results. The initial silver content in each fabric was normalized with respect to the fabric dry weight.

### Measurement of silver released into artificial sweat

Four different formulations of artificial sweat were prepared according to the International Standard Organization (ISO105-E04-2008E), American Association of Textile Chemists and Colorists (AATCC Test Method 15-2002) and the British Standard (BS EN1811-1999), as shown in Table 1, giving a pH range between 4.3 and 8.0. A weighed portion (0.20 - 0.30 g) of each fabric was soaked in artificial sweat at a ratio of 1:50 (w/v) for commercial fabrics and at a ratio of 1:100 (w/v) for laboratory-prepared fabrics. The use of different ratios between these fabrics was based on the initial amount of silver contained in the fabrics. The samples were incubated in a water bath at 37°C for 24 h. At the end of incubation period, the artificial sweat was collected and subjected to GFAAS analysis. The results were calculated as the total amount of silver released into the artificial sweat and normalized with respect to the dry weight of the fabric sample.

**Table 1:** Chemical composition of artificial sweat

Chemical composition	Concentration (% (w/v))			
	
	AATCC pH 4.3	ISO pH 5.5	ISO pH 8.0	EN pH 6.5
L-histidine monohydrochloride monohydrate (C_6_H_9_O_2_N_3_•HCl•H_2_O)	0.025	0.05	0.05	-
Sodium chloride (NaCl)	1.00	0.50	0.50	1.08
Disodium hydrogen orthophosphate dodecahydrate (Na_2_HPO_4_•12H_2_O)	-	-	0.50	-
Sodium dihydrogen orthophosphate dihydrate (NaH_2_PO_4_•2H_2_O)	-	0.22	-	-
Disodium hydrogen orthophosphate anhydrous (Na_2_HPO_4_)	0.10	-	-	-
Lactic acid (88%)	0.097	-	-	0.12
Urea	-	-	-	0.13

### Measurement of antibacterial properties

The antibacterial properties of the fabrics were determined according to the method of AATCC (Test Method 100-2004) with some modifications. *Escherichia coli *ATCC No. 25922 and *Staphylococcus aureus *ATCC No. 6538 were used as model Gram-negative and Gram-positive bacteria, respectively, to evaluate the antibacterial properties of the fabrics. The bacteria were suspended at a known initial concentration, within the approximate initial concentration of 4 × 10^5 ^colony forming units per milliliter (cfu/mL), was added to the 4.8 cm-diameter fabric samples and incubated with 1 ml TSB in a volumetric flask at 37°C for 24 h. At the end of the incubation period, the samples were quenched with 100 ml distilled water. The eluents were then serially diluted with 0.85% normal saline solution, each dilution being plated onto a separate petri dish containing TSA using an Automated Spiral Plating System (Autoplate 4000, Spiral Biotech, Norwood, MA) and further incubated at 37°C for 24 h. Viable bacteria were counted using an automatic colony counter (Flash & Go, IUL Instruments, Spain). The percentage reduction in the bacteria was calculated by the following formula:

Where A is the cfu of bacteria recovered from the inoculated fabric samples incubated for 24 h, and B is the cfu of bacteria recovered from the inoculated non-silver coated sample A0 incubated for 24 h.

## Results

### Characterization of silver particles in the silver suspension and fabric samples

**Figure 1:**
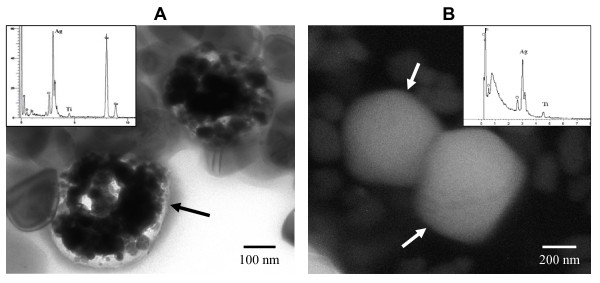
**Characterization of silver particles in the silver suspension**. **A) **Representative TEM image focused at the agglomerate of nano-sized particles contained in the suspension (black arrow). EDX analysis demonstrates a high peak of silver (small panel). **B) **Representative SEM image focused at spherical particles similar to that in the TEM image (white arrows). EDX analysis demonstrates a high peak of silver (small panel).

**Figure 2:**
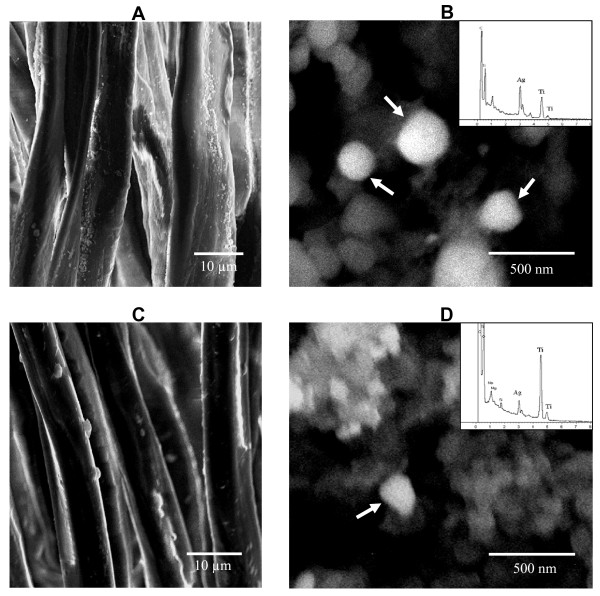
**Characterization of silver particles in the fabrics**. Representative SEM images show small particles attached onto the fibers of the laboratory-prepared fabric A4 **(A) **and the commercial fabric E **(C)**. Spherical particles (white arrows) in the ashes from fabric A4 **(B) **and fabric E **(D) **were detected. EDX analysis show peaks of silver and titanium (small panels).

In this study, the silver suspension used in laboratory-prepared fabrics (samples A1, A2, A3 and A4) was subjected to TEM and SEM analyses, both with EDX for characterization. From the TEM analysis, agglomerations of nano-sized particles were clearly visible (Figure 1A), and EDX analysis indicated that these agglomerations were predominantly silver, although a small peak of titanium was also observed. From the SEM analysis, white spherical-shaped particles with an approximate size of 500 nm were observed and were responsible for a high peak of silver (Figure 1B). The suspension was also analyzed by SEM-EDX mapping, which revealed the random distributed mixture of silver chloride and titanium dioxide. The XRD analysis indicated the rutile morphological structure of titanium dioxide (data not shown). One of each of the laboratory-prepared and commercial fabrics (samples A4 and E, respectively) was selected to confirm the presence of silver particles. At low magnification, SEM results showed a number of particles attached onto the fibers of both fabric samples (Figures 2A and 2C). Because of the difficulty to detect silver particles on the certain area of these fibers, the samples were then burned and the ashes were then subjected to SEM and EDX analyses. SEM analyses of the ashes revealed several spherical-shaped particles similar to that in silver suspension but with an approximate diameter of 200 nm (Figures 2B and 2D). In addition, EDX peaks of silver were observed in these samples.

### Antibacterial properties of fabric samples

**Figure 3:**
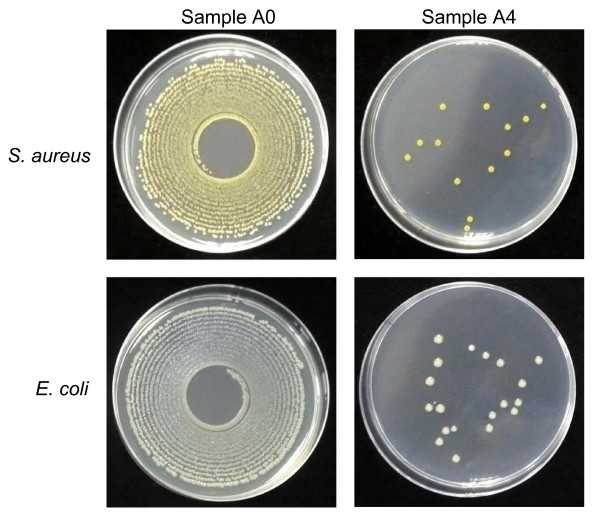
**Antibacterial properties of the fabrics shown on total agar plate counts**. The fabric samples, A0 and A4, were incubated in the presence of *S. aureus *and *E. coli *before plating and then incubating the agar plates for 24 h. The number of bacterial colonies that appear on the agar plate, relative to the control, reflects the antimicrobial (bacteriostatic and mainly bactericidal) properties of the fabric samples. The colonies were counted and the percent reduction of viable (proliferation competent) bacteria was calculated (Table 2).

To investigate the potential antibacterial properties, all fabric samples were subjected to an antibacterial assay against S. aureus and E. coli as model Gram-positive and Gram-negative bacteria, respectively. After incubation at 37°C for 24 h, most fabrics, except samples D and E, revealed a strong reduction in the number of S. aureus bacterial colonies, at higher than 98% inhibition of growth compared to the control sample (A0) and is summarized in Table 2. In contrast, only samples A4, E, F and G reduced the number of E. coli. Furthermore, sample E reduced the level of proliferation competent E. coli by only 29%. Samples A4, F and G, therefore, were the only samples to offer a high antibacterial activity against both organisms. As representative examples, the bacterial colonies of *S. aureus *and *E. coli *in agar plates after incubation with either the control sample A0 or the nanosilver treated sample A4 are demonstrated in Figure 3.

**Table 2:** Antibacterial effects of fabric samples

Sample	Percent reduction of bacteria	
	
	*S. aureus*	*E. coli*
A0	-	-
A1	98.04	-
A2	99.02	-
A3	97.30	-
A4	99.83	99.93
B	98.23	-
C	98.56	-
D	-	-
E	-	28.73
F	99.85	99.80
G	99.99	81.44

Data are mean of three independent experiments

### Release of silver from fabric samples into artificial sweat

The initial amount of silver in the fabrics was determined by acid digestion and subsequent GFAAS analyses, with the results being shown in the second column of Table 3. From the laboratory-prepared fabrics (samples A1, A2, A3 and A4), the amount of silver ranged from about 36 mg/kg to 425 mg/kg in relation to the concentrations of silver suspension used to coat these fabrics. Note that the uncoated control fabric (A0) revealed no detectable silver, as expected. From the commercial fabrics (samples B, C, D, E, F and G), silver was found in only three out of the six samples, which all were claimed to be "nanosilver shirts" by the manufacturers. Rather silver was not detected in samples B, C and D, as measured by both GFAAS and ICP. In addition, the initial amount of silver in samples E, F and G were somewhat low, with levels of about 15.2, 1.2 and 1.0 mg/kg, respectively.

**Table 3:** Initial silver content and total silver release in standard formulas of artificial sweat for 24 h

Sample	Initial silver content (mg/kg)	Silver released in artificial sweat (mg/kg)			
		
		AATCC Ph 4.3	ISO Ph 5.5	ISO Ph 8.0	EN Ph 6.5
A0	n.d.	n.d.	n.d.	n.d.	n.d.
A1	36.12 ± 22.42	21.01 ± 4.13	15.53 ± 3.62	34.27 ± 2.88	35.83 ± 19.68
A2	56.57 ± 34.28	33.39 ± 15.80	28.81 ± 10.34	66.54 ± 46.29	77.96 ± 23.80
A3	95.12 ± 33.12	70.15 ± 37.29	72.69 ± 11.99	82.22 ± 26.99	152.20 ± 36.54
A4	425.21 ± 93.73	217.61 ± 81.32	177.13 ± 57.13	268.31 ± 131.15	322.21 ± 87.00
B	n.d.	n.d.	n.d.	n.d.	n.d.
C	n.d.	n.d.	n.d.	n.d.	n.d.
D	n.d.	n.d.	n.d.	n.d.	n.d.
E	15.16 ± 9.90	0.08 ± 0.05	0.01 ± 0.01	0.50 ± 0.30	0.36 ± 0.10
F	1.22 ± 0.87	n.d.	n.d.	n.d.	0.05 ± 0.00
G	0.99 ± 1.53	n.d.	n.d.	n.d.	n.d.

Data are mean ± SD of three independent experiments.

n.d. = not detected

Small pieces of each fabric sample were subjected to immersion in one of the four standard formulations of artificial sweat (AATCC, ISO and EN with pH values of 4.3, 5.5, 6.5 and 8.0) at 37°C for 24 h. The amount of silver released from the fabrics into these artificial sweat formulations under these conditions are shown in Table 3. From the laboratory-prepared fabrics, the amount of silver released into each artificial sweat formulation was likely dependent on the initial amount of silver used in the treatment of the fabric, which ranged from about 15 mg/kg to 322 mg/kg. The highest level of released silver was found in sample A4 at pH 6.5 (EN artificial sweat formula), while the lowest level was found in sample A1 at pH 5.5 (ISO artificial sweat formula). From the commercial fabric samples B, C, D and G, silver could not be detected in any formulations of artificial sweat. This is of no surprise for fabric samples B, C and D, since no silver was detected in the fabric anyway. Whereas, silver was released from sample E into all formulations of artificial sweat at levels ranging from about 0.01 mg/kg to 0.5 mg/kg, depending upon the sweat formulation. In addition, a lower level of silver was released from sample F into the EN artificial sweat formula at pH 6.5.

## Discussion

Silver has long been used as an antibacterial agent for the treatment of many infectious diseases in humans, since the time before the emergence of antibiotics [22]. The mechanisms of silver's antibacterial properties were suggested to result from its binding to the bacterial cell wall and cell membrane and from the interaction with the thiol groups of bacterial proteins leading to their subsequent inactivation and loss of biochemical competence, without repair of which the cell will die [23]. The small size of silver nanoparticles gives improved antibacterial effects due to the increase in their surface area for interaction with the microorganisms [24], as well as potentially enhanced oxidation-solvation and uptake rates across cell membranes into the cytosol to disrupt intracellular protein thiol groups. Application of silver nanoparticles in textile materials is one of the most interesting applications to improve the quality of the products, including wound dressings and anti-bacterial clothes. Meanwhile, there is also increasing concern on the safety of these nanoproducts. To date there has not been any report on how much consumers may be exposed to silver particles during application. Therefore, artificial sweat was used as a model to study the release of silver from manufacturer-claimed nanosilver shirts as well as our laboratory-prepared nanosilver fabrics.

Firstly, the availability of silver in the silver suspension and in each fabric was characterized. From the product's information, the suspension contains silver chloride and titanium dioxide. We focused on the agglomeration of nano-sized particles in the TEM image where a high EDX peak of Ag and a small peak of Ti were demonstrated (Figure 1A). SEM analysis demonstrated agglomerated particles similar to that found in the TEM images, and with a similar EDX pattern with peaks of Ag and Ti (Figure 1B). These evidences support that the silver suspension used in this study contains silver nanoparticles. Agglomeration is the natural phenomenon of colloidal nanoparticles due to their ultrafine size [25]. In the fabric ashes, silver particles detected from the laboratory-prepared fabric A4 (Figure 2B) were similar in their size to that from fabric E (Figure 2D), but smaller than that of their original form in the silver suspension (Figure 1B). It was likely that the burning process reduced the silver particle size agglomeration. The height of the Ag peak in the EDX graph indicated the amount of silver found in the focused area of SEM image. Besides silver, other compounds were also obtained in EDX peaks, the most prominent of which was titanium (Figures 2B and 2D).

Titanium is one of the frequently used nanomaterials in consumer products, including cosmetics, sunscreens and textiles [2]. The composition of the silver solution used in this study also contained titanium dioxide which, in the presence of UV light, has the potential to kill bacteria [26]. However, some forms of titanium dioxide, such as nitrogen- and carbon-doped titanium dioxide, are also effective under visible light [27]. Hence, titanium dioxide is usually incorporated into most antibacterial materials where it exerts a synergistic antibacterial effect when combined with silver [28,29]. In addition, titanium dioxide can reduce the use of silver, with the advantages of an increased whiteness and a reduced cost of the products.

Since the main purpose of fabric samples is to prevent bacteria, the antibacterial effect of the different fabrics was evaluated using S. aureus and E. coli as model Gram-positive and Gram-negative bacteria, respectively. Most of the fabrics used in this study showed a higher antibacterial activity against *S. aureus *than that against *E. coli *(Table 2). The mechanism underlying this antibacterial activity for each of the fabrics was not investigated, but it is likely to not be solely due to nanosilver particles as those fabrics which had silver levels below the detection levels (fabrics B and C) showed essentially as good an antibacterial activity against *S. aureus *as the silver impregnated fabrics. Perhaps, this then is accounted for by the titanium dioxide. However, the differences between the inhibition of *S. aureus *and *E. coli *growth may result from the difference in the compositions of the bacterial cell walls where the cell wall of Gram-negative, but not Gram-positive, bacteria consists of lipopolysaccharides which may provide a more effective protection against bactericides [30]. Certainly, further screening of more representatives of each bacterial division is required to support such a notion.

By using the coating procedures, it was likely that a 10 g/L silver suspension was sufficient for preparing antibacterial fabrics. At this treatment concentration, the fabrics induced a 99.83% and 99.93% reduction of proliferation competency in *S. aureus *and *E. coli*, respectively. However, for commercial fabrics, there was no correlation between the initial level of silver in the fabrics and their antibacterial properties, perhaps due to the compounding action of other components such as titanium dioxide. In this light, it is worth noting that fabrics B and C had no detectable silver but could significantly inhibit *S. aureus *proliferation competency. In addition, the antibacterial properties of silver nanoparticles can be varied by their size, shape and surface modifications [3,24,31]. These factors might partly explain the uncorrelated results between amount of silver found in the fabrics and their antibacterial properties.

Artificial sweat has been used frequently for assessing the level of metal release from various materials, such as to study nickel released from earrings, which shows a relationship to dermatitis [32]. However, the composition of sweat not only varies between individuals, but also within an individual according to their body region, age, season, degree of acclimation, diet, infection status and level of activity [33]. Therefore, the effect of variations in the artificial sweat composition on the silver leaching levels was evaluated using the four international standard artificial sweat formulations (Table 3).

Even though the manufacturers claimed nanosilver products, the data indicated that silver was detected only in three out of six commercial fabrics. One of the possibilities might be due to the very low amount of silver available in the products, less than the lower threshold detection limit (0.26 μg/L) of the instrument. In addition, as the level of silver approaches the detection limit, the less accurate are the measured results. Regardless, for those fabrics with detectable levels of silver, it was observed that silver was released into the artificial sweat after incubation at 37°C for 24 h.

For the laboratory-prepared fabrics, the amount of silver released into the artificial sweat was dependent upon the initial amount of silver coated onto each sample. However, nanosilver fabrics can be prepared by several methods [5,6,34]. In this study, some commercial fabrics had different textures and thus likely methods of incorporating silver into them. For instance, unlike the other commercial fabrics, fabric E was prepared by incorporating silver nanoparticles into the fibers (pers. com. with the manufacture). Variation in the fabric quality might be one of the factors that affected the release of silver into artificial sweat.

The ISO artificial sweat formulation (pH 5.5) showed the lowest detected levels of silver leaching from both laboratory-made and commercially obtained fabrics. This pH is similar to the pH of normal human skin [33]. In contrast, in the EN artificial sweat formulation (pH 6.5), the largest release of silver from all fabrics was observed. This standard formulation is normally used for determining metal release from jewelry [20,21]. As shown in Table 1, it contained urea, which was not included in the other artificial sweat formulations used in this study. Therefore, the release of silver from the fabrics was also likely to be affected by the pH and formulations of artificial sweat.

Several lines of nanosilver-based textile fabrics are already on the market. Dermal exposure represents an important potential route of exposure for these nanoparticles. Yet, data in relation to the potential human health risk are very rare. The release of nanoparticles from the fabrics under various conditions, such as sweating, repetitive attrition and laundering, are considered essential information. In this study, it is possible that artificial sweat might facilitate transfer of silver-nanoparticle-treated fabric to the skin surface [35]. However, it remains unclear whether these nanoparticles released from the products at the site of application were absorbed into the body. Close contact may allow nanoparticles to penetrate through compromised skin barrier and gain access to the dermal capillaries [12]. With regard to this notion, abnormal elevation of blood silver levels, Argyria-like symptoms and hepatotoxicity following the use of nanosilver coated dressings for burns in clinical application has been reported [36]. Our investigation may have a significant advantage for the evaluation of the human health risk of silver nanoparticles released from textile products. Future advanced research in relation to these aspects is needed to be conducted.

## Conclusions

This study provides the unprecedented data on detecting silver released from antibacterial fabric products using artificial sweat as a model to represent the human skin environment. Silver was detected in some commercial fabrics which claimed to be nanosilver shirts. The amount of silver released from the fabrics into artificial sweat was dependent upon the initial amount of silver coating, the fabric quality, pH and artificial sweat formulations. This information might be useful to evaluate potential human risk when exposed to silver nanoparticles form textile materials.

## Competing interests

The authors declare that they have no competing interests.

## Authors' contributions

KK participated in the design of the study, performed the particle characterization, silver release study and antibacterial assay, and drafted the manuscript. SS participated in the design of silver release study. KB carried out laboratory fabric preparation. WK participated in the design of the laboratory fabric preparation. RM conceived the study and participated in its design and coordination and helped to draft the manuscript. All authors read and approved the final manuscript.
